# Detection and Alterations of Acetylcarnitine in Human Skeletal Muscles by 1H MRS at 7 T

**DOI:** 10.1097/RLI.0000000000000355

**Published:** 2017-02-10

**Authors:** Radka Klepochová, Ladislav Valkovič, Martin Gajdošík, Thomas Hochwartner, Harald Tschan, Michael Krebs, Siegfried Trattnig, Martin Krššák

**Affiliations:** From the *High-Field MR Center, Department of Biomedical Imaging and Image-Guided Therapy, Medical University of Vienna, Vienna, Austria; †Department of NMR Spectroscopy and Mass Spectrometry, Faculty of Chemical and Food Technology, Slovak University of Technology, Bratislava, Slovakia; ‡Christian Doppler Laboratory for Clinical Molecular MR Imaging, Vienna, Austria; §University of Oxford Centre for Clinical Magnetic Resonance Research (OCMR), University of Oxford, Oxford, United Kingdom; ∥Department of Imaging Methods, Institute of Measurements Science, Slovak Academy of Sciences, Bratislava, Slovakia; ¶Division of Endocrinology and Metabolism, Department of Internal Medicine III, Medical University of Vienna; and #Centre of Sport Science and University Sport, University of Vienna, Vienna, Austria.

**Keywords:** acetylcarnitine, ^1^H MRS, long TE, skeletal muscles, 7 T

## Abstract

**Objectives:**

The aims of this study were to detect the acetylcarnitine resonance line at 2.13 ppm in the human vastus lateralis and soleus muscles, assess T_1_ and T_2_ relaxation times, and investigate the diurnal and exercise-related changes in absolute concentration noninvasively, using proton magnetic resonance spectroscopy at 7 T.

**Materials and Methods:**

All measurements were performed on a 7 T whole-body Magnetom MR system with a 28-channel knee coil. Five healthy, moderately trained volunteers participated in the assessment of the detectability, repeatability, and relaxation times of acetylcarnitine. For the evaluation of the effect of training status, another 5 healthy, normally active volunteers were examined. In addition, normally active volunteers underwent a day-long protocol to estimate diurnal changes and response to the exercise.

**Results:**

Using a long echo time of 350 milliseconds, we were able to detect the acetylcarnitine resonance line at 2.13 ppm in both muscle groups without significant lipid contamination. The T_1_ of acetylcarnitine in the vastus lateralis muscle was found to be 1807.2 ± 513.1 milliseconds and T_2_ was found to be 129.9 ± 44.9 milliseconds. Concentrations of acetylcarnitine from the vastus lateralis muscle in moderately trained volunteers were higher than concentrations from normally active volunteers. Acetylcarnitine concentrations changed during the day, tending to be higher in the morning after an overnight fast than after lunch. After 10 minutes of high-intensity exercise, the concentration significantly increased, and 15 minutes after cessation of exercise, a decrease could be observed.

**Conclusions:**

Our results demonstrate an effective detection of acetylcarnitine using a long TE of 350 milliseconds at 7 T in the vastus lateralis and soleus muscles with high repeatability and reliability on a 7 T scanner. Our data emphasize the need for strict standardization, physical activity, and dietary conditions for the measurement of the acetylcarnitine.

The biologically active enantiomer l-carnitine (hereafter “carnitine”) is a compound known for its important function in fat metabolism and exists as free carnitine or in esterified short- and long-chain forms. Acetylcarnitine is predominantly the short-chain form of carnitine (Fig. 1).^1^ Carnitine synthesized in the liver and kidney^2^ is released and taken up by other tissues. The main reservoir for the whole-body store of carnitine is skeletal muscle (at 98%; of this amount, approximately 80% is in the form of carnitine and 20% is in the form of acetylcarnitine).^1^

**FIGURE 1:**
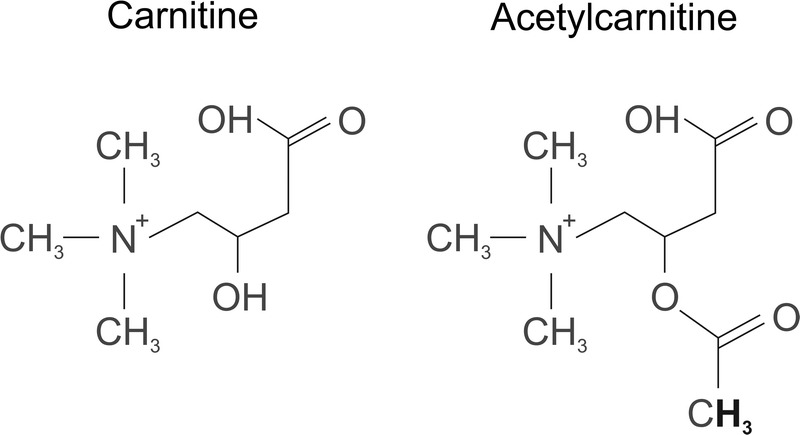
Structure of carnitine and acetylcarnitine. In the acetylcarnitine molecule, protons contributing to the resonance at 2.13 ppm in the ^1^H MRS spectra are highlighted.

Carnitine has 2 main functions. First is the translocation of long-chain fatty acids from cytosol to the mitochondrial matrix,^3^ and the second involves the formation of acetylcarnitine from short-chain acetyl-coenzyme A and protection against acetyl-coenzyme A accumulation, which is potentially harmful to cellular function.^4^ In addition, a low acetyl-coenzyme A/free coenzyme A ratio is needed to maintain pyruvate dehydrogenation activity.^5^ Low carnitine concentrations may reduce or inhibit pyruvate dehydrogenation activity and, therefore, reduce the oxidative degradation of glucose. Moreover, low carnitine concentrations cause a reduction of the fatty acid transport into the mitochondria, which results in the cytosolic accumulation of triglycerides.^6^ Impaired pyruvate dehydrogenation activity and an increase in intramuscular triglyceride is implicated in the pathogenesis of insulin resistance, thereby contributing to the development of type 2 diabetes mellitus.^6–8^

For many years, biochemical analysis of tissue biopsies was the only method capable of detecting carnitine and acetylcarnitine concentrations in tissues of interest. A noninvasive alternative to this procedure would enable broader physiological research. Proton magnetic resonance spectroscopy (^1^H MRS) has been proposed and validated for the measurement of skeletal muscle carnitine and acetylcarnitine concentrations at lower field strengths.^9–11^

Aside from the ^1^H MR resonance lines of creatine (3.03 and 3.9 ppm) and the complex lines of intramyocellular and extramyocellular lipids (0.9 to 1–5 ppm, as well as 2.2–2.4 ppm), the main spectral lines of carnitine that originate from the acetyl group of acetylcarnitine at the 2.13 ppm chemical shift and the trimethyl ammonium (TMA) group from carnitine and acetylcarnitine at the 3.20 ppm chemical shift^9,10,12^ can be detected by ^1^H-MRS. The straight forward detection and quantification is challenging, due to the strong overlap of the acetylcarnitine line with lipid resonances at 2.13 ppm, under in vivo conditions, and the fact that the line at 3.20 ppm represents a combination of the TMA groups of carnitine, acetylcarnitine, and choline. As acetylcarnitine metabolism adapts to an exercise challenge, the detectability of acetylcarnitine is improved immediately after a workout, when concentrations are increased and the resonance lines are well-resolved.^12^ Recently, the differences in T_2_ and T_1_ relaxation times of acetylcarnitine and lipids have been employed for the detection of the 2.13 ppm line in long TE or T_1_-edited ^1^H MRS of the quadriceps muscle at rest at 3 T.^9,11^ In addition, the recent reports point toward different concentrations of acetylcarnitine in different muscle groups. Higher concentration values in the vastus lateralis (VL)^9^ allow detection under resting conditions.

Recently introduced whole-body MR systems operating at a field strength of 7 T brought the advantage of increased signal-to-noise ratio (SNR) and improved spectral resolution in the field of skeletal muscle MR spectroscopy. Usually, this can be translated into smaller volumes of interest (VOIs), and thus, enhanced morphological precision, decreased acquisition times, or improved detection of low metabolic concentrations.^13–16^

Thus, in this work, we aimed to detect the acetylcarnitine resonance line at 2.13 ppm in the thigh muscle (VL) and calf muscle (soleus) using long TE MRS at 7 T, which, in addition to increased SNR, should provide enhanced spectral resolution and, therefore, improve the repeatability of the measurements. For the quantification of the molar concentration of acetylcarnitine, we first assessed metabolite T_1_ and T_2_ relaxation times at 7 T. Since acetylcarnitine concentrations in plasma are known to vary during the day^17^ and increase after high-intensity exercise,^18–20^ we also aimed to investigate the diurnal and exercise-related changes in the concentration of acetylcarnitine in skeletal muscle noninvasively, using ^1^H MRS at 7 T. For our purposes, we called these variations diurnal changes, which include all changes during the day, that is, overnight fasting, feeding, and exercise.

## MATERIALS AND METHODS

### Study Population

Ten healthy, active volunteers (8 males, 2 females) participated in the study. Written informed consent was provided in accordance with the local ethics committee requirements.

For the determination of maximal oxygen uptake (VO_2max_), all participants underwent a standardized protocol with continuous increments, until exhaustion, on a cycle ergometer (Lode Excalibur, Groningen, the Netherlands). Measurement of VO_2max_ and other parameters was performed via “breath-by-breath” Spiroergometry (Master CPX, VIASYS Healthcare). Based on the self-reported physical activity and measured maximal oxygen uptake (VO_2max_), volunteers were divided into 2 groups, the moderately trained (4 or more training units a week, VO_2max_ ≥ 33.0 mL · min^−1^ · kg^−1^ for females, and VO_2max_ ≥ 45.0 mL · min^−1^ · kg^−1^ for males) and the normally active (1 to 3 training units a week, VO_2max_ ≤ 33.0 mL · min^−1^ · kg^−1^ for females, and VO_2max_ ≤ 45.0 mL · min^−1^ · kg^−1^ for males). Forty-five to 90 minutes of continuous exercise, regardless of endurance or resistive nature, was considered 1 training unit.

### Magnetic Resonance Measurements

All MR measurements were performed on a 7 T whole-body Magnetom MR system (Siemens Healthineers, Erlangen, Germany). A 28-channel knee coil (QED, Mayfield Village, OH) was used to acquire spectra from the quadriceps and soleus (SOL) muscle of the left leg. All volunteers were examined in the supine position.

### Detectability, Repeatability, and Relaxation Times of Acetylcarnitine

Of the whole study group, 5 healthy, moderately trained volunteers (age 29.8 ± 4.2 years, body mass index 23.0 ± 0.6 kg/m^2^, self-reported activity of 4 to 7 training units a week, mean VO_2max_ 45.5 ± 6.6 mL · min^−1^ · kg^−1^) participated in this part of the study. All measurements for repeatability were conducted in the morning hours and measurements for the assessment of relaxation times were conducted in the afternoon (2 to 3 hours after lunch). Volunteers did not perform any physical exercise on the day of the measurement and 1 day before.

#### Detectability

T_1_-weighted multislice images were acquired and used for VOI positioning. Spatial selection was achieved using a STEAM localization sequence (TR/TE, 2000/350 milliseconds; spectral bandwidth, 3 kHz; number of averages, 128; delta frequency, −2.5 ppm relative to water resonance; number of preparation scans, 4) and the VOI (40 × 35 × 15 mm^3^) was carefully placed in the VL muscle or in the SOL muscle. Localized shimming was performed manually, on the adjustment volume that matched the VOI, after automatic field-map acquisition based on gradient recalled, double echo field-map acquisition (GRE-SHIM; Siemens Healthcare, Erlangen, Germany). The final linewidth of water was in the range of 28 to 38 Hz in magnitude mode.

For concentration determination, the water signal was measured separately (TR/TE, 2000/20 milliseconds; number of averages, 1; delta frequency, 0 ppm).

In 1 volunteer, an additional spectrum with a short echo time of 50 milliseconds was acquired to demonstrate acetylcarnitine-lipid overlap in the observed spectral region.

#### Repeatability

Test-retest measurements were performed in the morning hours in the VL muscle to determine the repeatability of the protocol. Before the measurements, the position of thigh in the coil had been marked. Parameters for measurement, size of VOI, shimming, and measurement of the water signal were as described previously. The retest measurements were performed after a short break during, which the volunteers were taken out of the magnet and walked for approximately 1 minute in the scanner room. Full, careful repositioning and shimming procedures were performed with the same measurement protocol. To assess the repeatability of acetylcarnitine, the mean coefficient of variation was calculated for test-retest measurements.

#### T_2_ Relaxation Time

For the assessment of the T_2_ relaxation time of the acetylcarnitine spectral line at 2.13 ppm, a series of 8 spectra with 8 different TEs (100,150,200,250,300,350,400, and 450 milliseconds) were acquired in both muscles in all 5 volunteers. Other sequence parameters and VOIs remained the same as for detectability. Total measurement time was approximately 45 minutes.

#### T_1_ Relaxation Time

The T_1_ relaxation time was measured in the VL only, using an inversion recovery sequence with the following parameters: TR, 6000 milliseconds; TE, 350 milliseconds; and TI of 20, 200, 500, 800, 2000, 5000 milliseconds; number of averages, 128; delta frequency, −2.5 ppm (used for all RF-pulses); preparation scans, 4; inversion pulse (WURST) duration, 5000 μs. As a long TE was used, no water suppression was necessary. Total measurement time was approximately 1 hour 30 minutes.

### Diurnal Changes and Response to Exercise

**FIGURE 2:**
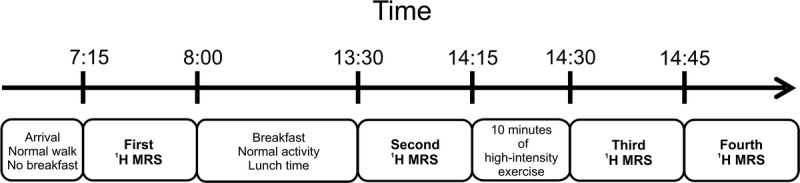
Schematic illustration of experimental design, including time points of measurements.

The second, normally active group of volunteers (age 29.2 ± 1.3 years, body mass index 22.8 ± 2.5 kg/m^2^, self-reported activity of 1 to 3 training units a week, mean VO_2max_ 39.9 ± 7.3 mL · min^−1^ · kg^−1^) participated in the second part of this study. All of them underwent the first measurement early in the morning (7 am), after an overnight fast and without strenuous exercise in the morning. Data were obtained from both the VL and the SOL muscles using a STEAM sequence with the same protocol as described previously. The second measurement of acetylcarnitine from the same VOIs was performed immediately after a normal hospital canteen lunch (~6 hours after the first scan). Subsequently, each volunteer performed high-intensity exercise (10 minutes of continuous squats; n = 274 ± 38). The ^1^H MRS measurements, performed in the VL only, were repeated twice after the exercise (starting at 0 and 15 minutes). A schematic illustration of the experimental design, including the time points of ^1^H MRS, is shown in Figure 2.

Data from the second measurement, that is, after lunch, were used also to compare the afternoon concentrations of acetylcarnitine in moderately trained and normally active volunteers.

### Spectral Processing, Assessment of Relaxation Times, and Absolute Quantification

All spectra were fitted using the Advanced Method for Accurate, Robust, and Efficient Spectral (AMARES) fitting algorithm in the jMRUI v5.2 software^21^ with spectral lines of acetylcarnitine, creatine, TMA, lipids, and water modeled as single Lorentzians. Lipids surrounding the acetylcarnitine peak were fitted with a constrained frequency of 2.0 to 2.1 ppm and 2.17 to 2.30 ppm to avoid their influence on fitted acetylcarnitine.

Relaxation times were calculated in MATLAB by fitting the data to the mono-exponential functions, M_TE_ = M_0_.e^(−TE/T2)^ and M_TR_ = M_0_.(1-b.e^(−TI/T1)^). Although full inversion (ie, b = 2) could be expected, we used a 3-parameter fitting routine^22^ (ie, b-parameter is free), which is often more robust.

Using the water peak as an internal reference, the concentration of acetylcarnitine was calculated according to the formula for millimolar concentration in wet weight (mmol/kg ww):

**Formula:**
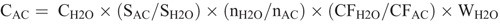


where S is the signal intensities of metabolites (H_2_O, water; AC, acetylcarnitine), n is the number of protons in a water and acetylcarnitine molecule, CF is correction factors for T_1_ and T_2_ relaxations, C_H2O_ = 55,556 mmol/L is the concentration of the water, and W_H2O_ is the approximate water content of skeletal muscle tissue, that is, 0.77 L/kg wet weight of the tissue.

Differences in the values of the acetylcarnitine concentration during diurnal changes and after exercise were tested for significance by repeated-measures analysis of variance and Fisher least significant difference–corrected post hoc test. Differences between groups and between muscles were tested for significance by 1-way analysis of variance and least significant difference–corrected post hoc tests. All analyses were done in SPSS (version 21.0; IBM SPSS, Chicago, IL). All values are provided as mean ± SD, and a *P* value less than 0.05 was considered significant.

## RESULTS

### Detectability and Repeatability of Acetylcarnitine in the VL and SOL Muscles Under Resting Conditions

**FIGURE 3:**
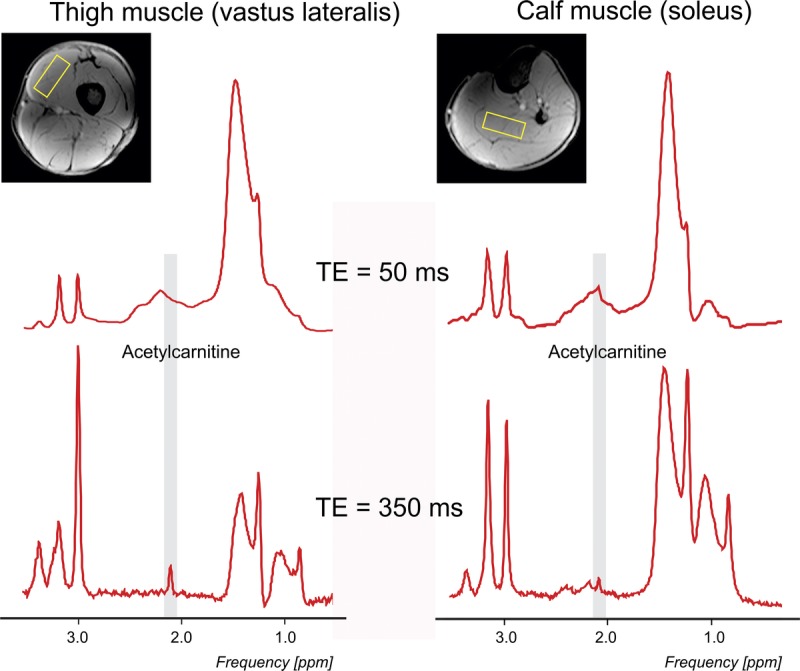
Comparison of short (50 milliseconds) and long (350 milliseconds) TE spectra from the VL and the SOL muscles (left leg). T_1_-weighted axial images and voxel placements are inserted. For better visualization, a 1.5-Hz Lorentzian apodization filter was applied to reduce noise in the spectra.

Using a long TE, we were able to detect the acetylcarnitine resonance line at 2.13 ppm (Fig. 3) in both muscle groups in all volunteers without significant lipid contamination.

The mean individual coefficient of variation estimated in 5 moderately trained volunteers by test-retest measurement was 11.6%.

### Relaxation Times of Acetylcarnitine

The T_1_ of acetylcarnitine in the VL muscle was found to be 1807.2 ± 513.1 milliseconds, and T_2_ was found to be 129.9 ± 44.9 milliseconds. T_1_ and T_2_ relaxation times of acetylcarnitine from the VL with the reported fitting precision and b values that demonstrate the inversion efficiency are shown in Table 1. Despite the fact that we recruited moderately trained volunteers in whom higher acetylcarnitine muscle levels were assumed, we were not able to resolve the acetylcarnitine peak in the SOL muscle spectra using a short TE, making quantification of T_2_ in the SOL impossible. Therefore, concentrations of acetylcarnitine from the SOL were corrected with relaxation coefficients from the VL muscle.

**TABLE 1:**
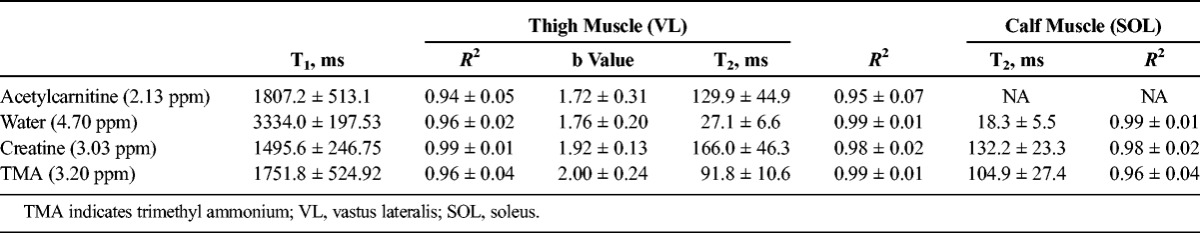
T_1_ and T_2_ Relaxation Times of Acetylcarnitine, Creatine, TMA, and Water + SD From VL Muscle at 7 T

### Absolute Quantification

The acetylcarnitine concentration was quantified in all 10, that is, moderately trained and normally active, volunteers in both muscles and from all measurements. ^1^H MRS measurements for this part of experiment were always performed in the afternoon under the same physiological conditions.

**FIGURE 4:**
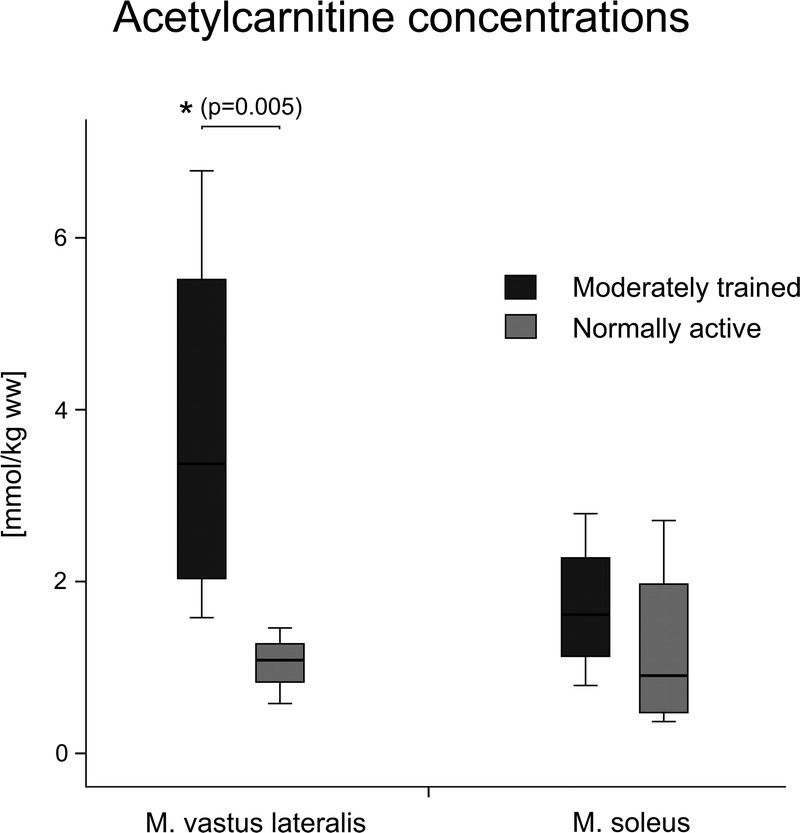
Box plots showing concentrations of acetylcarnitine from the VL and the SOL muscle ± SD of moderately trained and normally active volunteers measured in the afternoon. *Significant difference at the *P* < 0.05 level between the concentrations of acetylcarnitine in moderately trained and normally active volunteers in the VL muscle.

Concentrations of acetylcarnitine of moderately trained volunteers were significantly higher (3.83 ± 1.99 mmol/kg ww) than concentrations of normally active volunteers (1.29 ± 0.62 mmol/kg ww) (*P* = 0.005) in the VL muscle. In the SOL muscle, no significant differences were found between the 2 groups of volunteers. The concentration of acetylcarnitine in the SOL muscle of moderately trained volunteers was 1.70 ± 0.83 mmol/kg ww, and the concentration in normally active volunteers was 1.21 ± 0.92 mmol/kg ww. Moreover, in general, concentrations of acetylcarnitine in the VL muscle were higher than in the SOL muscle. More details are given in Figure 4.

### Diurnal Changes and Response to Exercise

**FIGURE 5:**
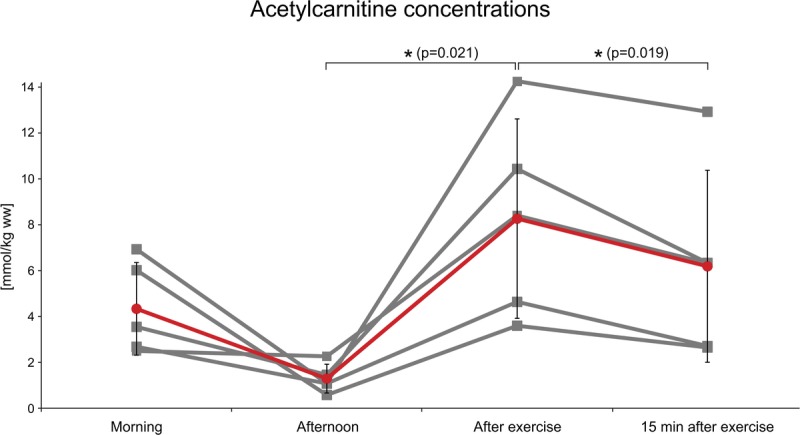
Line graphs depicting absolute concentrations of acetylcarnitine in the VL muscle in 5 normally active volunteers (gray lines) and mean (red line) from the morning, on an empty stomach, in the afternoon after lunch, after exercise, and 15 minutes after exercise. *Significant difference at the *P* < 0.05 level between the afternoon concentrations of acetylcarnitine and after high-intensity exercise concentrations in the VL muscle and between concentrations of acetylcarnitine after high-intensity exercise and 15 minutes after high-intensity exercise in the VL muscle in normally active volunteers.

**FIGURE 6:**
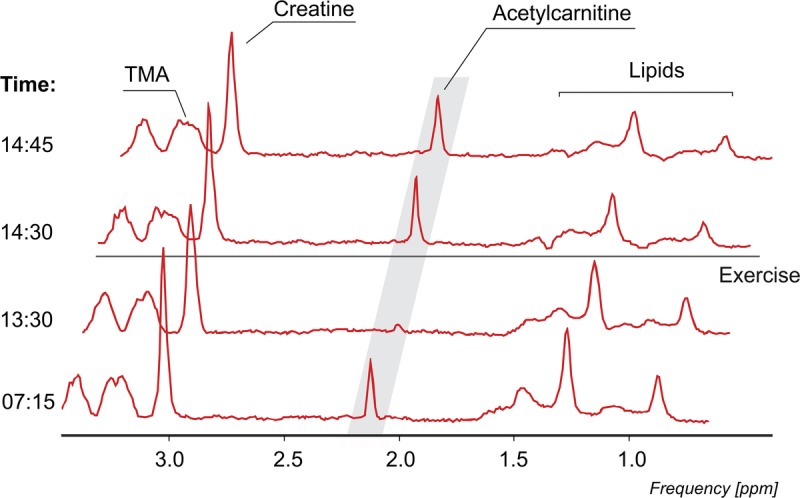
^1^H MRS spectra from the VL muscle acquired in the morning, afternoon, immediately after exercise, and 15 minutes after exercise (1.5 Hz Lorentzian apodization).

Individual and pooled results are depicted in Figure 5 and representative spectra from one volunteer in Figure 6. Acetylcarnitine concentrations changed during the day, tending to be higher in the morning (4.33 ± 0.9 mmol/kg ww) than after lunch (1.29 ± 0.62 mmol/kg ww). After 10 minutes of high-intensity exercise, the concentration significantly increased from 1.29 ± 0.62 to 8.26 ± 4.34 mmol/kg ww (*P* = 0.021), and, again, significantly decreased 15 minutes after cessation of the exercise, with concentrations of 8.26 ± 4.34 to 6.18 ± 4.18 mmol/kg ww (*P* = 0.019).

## DISCUSSION

Our study demonstrated that acetylcarnitine could be detected by ^1^H MRS at 7 T in 2 different and, from the point of view of physiological research, very important skeletal muscle groups, SOL and VL. Furthermore, we determined the relaxation times of acetylcarnitine in skeletal muscle at 7 T, which are essential for absolute quantification purposes. We have also followed the diurnal changes of acetylcarnitine concentrations, which were established from the biochemical analysis of the serum.^17^ We also measured skeletal muscle acetylcarnitine levels in normally active and moderately trained volunteers in the morning on an empty stomach and in the afternoon after lunch, as well as after 10 minutes of high-intensity physical workout.

### Detectability of Acetylcarnitine

The results of the long TE measurement revealed that acetylcarnitine appears as an obvious, distinguishable peak without significant lipid contamination. The echo time of 350 milliseconds was chosen here since the spectra with even longer TE 400 milliseconds and 450 milliseconds suffered a substantial overall loss in SNR. We applied a stimulated echo rather than a spin echo-based sequence as our 7 T standard technique for ^1^H MRS measurements due to the lower demands on RF coil efficiency, lower SAR deposition (no 180-degree pulses involved), and lower sensitivity to echo time phase evolution due to the J coupling.^23^ It is, however, true that a well-prepared/set PRESS sequence could be also used in the current setup.

Acetylcarnitine levels were measured in 2 skeletal muscle groups, the VL and the SOL, with higher concentrations of acetylcarnitine in the VL muscle than in the SOL muscle. According to the results obtained by biochemical analysis of biopsy in the study by Gollnick et al,^24^ there is an approximately equal proportion of slow-twitch fibers and fast-twitch fibers in the VL, whereas the SOL muscle contains predominantly slow-twitch–type fibers. Moreover, the activity of glycolytic enzymes were lower in slow-twitch fibers.^24^ Thus, we assume that different concentrations of acetylcarnitine between muscles may be caused by different fiber compositions and enzyme profiles. Based on the higher concentrations and larger dynamic range appears VL as a more suitable for future cross-sectional and interventional studies.

Acetylcarnitine concentrations were expected to be higher in endurance-trained athletes, possibly as a result of enhanced fat oxidation.^25^ It has been found that endurance exercise training increases fat oxidation and activates, among other processes, pyruvate dehydrogenation activity, leading to markedly increased mitochondrial generation of acetyl-coenzyme A and, secondarily, acetylcarnitine.^26^ In addition, carnitine acetyl coenzyme A transferase activity tasked with conversion of acetyl-coenzyme A into membrane-permeable acetylcarnitine was found to be significantly higher in the endurance-trained athletes.^9^ Process may be important in maintaining mitochondrial efflux of excess acetyl-coenzyme A that otherwise could inhibit pyruvate dehydrogenase.^25^ Moreover, the high-intensity exercise state is associated with a rapid change in muscle carnitine metabolism characterized by an increase in acetylcarnitine content and decreased free carnitine content.^19^ Although further studies are necessary to better understand whole-body acetylcarnitine physiology in humans.

In our study, 2 groups of volunteers were investigated, moderately trained and normally active. Moderately trained volunteers showed higher acetylcarnitine concentrations in the VL and, nonsignificantly, also in the SOL muscle than did normally active volunteers. This is in agreement with a previous study by Lindeboom et al,^9^ associating acetylcarnitine concentration with training status, increased insulin sensitivity, and higher in vivo mitochondrial function.

### Relaxation Times of Acetylcarnitine

To the best of our knowledge, the results presented here are the first in vivo assessed relaxation times of the 2.13 ppm acetylcarnitine resonance in the VL muscle at 7 T. Lindeboom et al^9^ assessed the spin-spin relaxation time of the 2.13 ppm acetylcarnitine resonance in the VL muscle at 3 T, which was longer (265 ± 45 milliseconds) than our results (129.9 ± 44 milliseconds), and is in good agreement with the theory of a T_2_ time decrease with the magnetic field strength. Ren et al,^12^ in their work, determined both spin-spin and spin-lattice relaxation times of a 3.17-ppm acetylcarnitine resonance line at 7 T in the SOL muscle. In resting muscle, they could not resolve the carnitine peak due to the low concentration; thus, T_1_ and T_2_ relaxation times were assessed in post-exercise spectra. The T_1_ values reported there, using an inversion recovery method for measurements, were shorter (900 ± 110 milliseconds) than reported here (1807 ± 513 milliseconds). These differences could be attributable to the different spectral line of acetylcarnitine (3.17 ppm vs 2.13 ppm) and the different muscle groups under examination (SOL vs VL).^12^ However, T_2_ values from their study were comparable to our findings in both muscles.^12^

### Diurnal Changes and Response to Exercise

Serum carnitine and acetylcarnitine concentrations were previously found to vary significantly during the day in reaction to plasma free fatty acid concentrations.^17^ According to the literature, free fatty acid concentrations are increased in the fasting state and acutely after exercise, and, on the other hand, decreased after a carbohydrate-rich meal.^27,28^ It is interesting that the results of repeated skeletal muscle acetylcarnitine measurements during the day mirror this time course. We performed ^1^H MRS measurements early in the morning after an overnight fast, and, subsequently, during the afternoon after lunch, and skeletal muscle acetylcarnitine concentrations in the morning were higher than in the afternoon after lunch. Previous findings showed that 10 minutes of high-intensity exercise can change the skeletal muscle carnitine pool^4^ and increase the formation of acetylcarnitine, leading to its subsequent accumulation.^29^ Thus, a high-intensity exercise challenge to the VL muscle by performing squats continuously for 10 minutes was applied to assess the effect of physical load on the concentration of acetylcarnitine in skeletal muscle. An increase in the acetylcarnitine level was detected in each of the 5 individuals. In accordance with the notion that acetylcarnitine varies with free fatty acid concentrations, increased plasma free fatty acid concentrations were observed after exhaustive exercise on a cycle ergometer.^28^ Approximately 15 minutes after the cessation of exercise, we could detect acetylcarnitine depletion or washout, which is in accordance with the findings of Seiler et al,^20^ on a trained group of subjects. For our purposes, we called these variations diurnal changes, which include all changes during the day, that is, overnight fasting, feeding, and exercise.

Nevertheless, from our results, we cannot draw general conclusions about exact diurnal changes and the exercise effects on skeletal acetylcarnitine concentration, as our study included only a limited number of subjects and was aimed mainly on the methodological aspects of acetylcarnitine detection and quantification in 2 different muscle groups of young healthy volunteers at an MR field strength of 7 T. Further studies on the influence of food intake, diet composition, sex, age, and different pathophysiological conditions with a larger number of volunteers are necessary.

## CONCLUSIONS

Our results demonstrate an effective detection of acetylcarnitine using a long TE of 350 milliseconds at 7 T in the VL and the SOL skeletal muscles. We were able to observe the relaxation behavior and calculate the T_1_ and T_2_ relaxation time values for resting VL muscle. We could also observe the diurnal changes of acetylcarnitine concentration and changes after high-intensity exercise. The differences in acetylcarnitine levels in skeletal muscle among different volunteers are linked to different training statuses and suggest an adaption of skeletal muscle metabolism to the training. Our data emphasize the need for strict standardization, physical activity, and dietary conditions for the measurement of acetylcarnitine/carnitine. Further studies can help clarify the role of acetylcarnitine and its relationship to the impaired mitochondrial oxidation of fatty acids and insulin resistance in skeletal muscle.
